# Effectiveness of Educational Intervention Based on Transtheoretical Model on Physical Activity and Menopausal Symptoms

**DOI:** 10.1155/2022/1791445

**Published:** 2022-12-13

**Authors:** Leila Fallahipour, Mahin Nazari, Masoud Karimi, Elahe Zare

**Affiliations:** Research Center for Health Sciences, Institute of Health, Department of Health Promotion, School of Health, Shiraz University of Medical Sciences, Shiraz, Iran

## Abstract

**Introduction:**

Menopause is an inevitable part of women's lives. Physical activity as nonhormonal therapy can decrease the symptoms of menopause. This study is aimed at investigating the effect of educational intervention based on the transtheoretical model (TTM) on physical activity and menopausal symptoms of female clients of the health center in Pasargad City, Iran.

**Methods:**

This quasi-experimental study was performed on 140 females admitted to health center of Pasargad City, Fars Province, Iran (intervention = 70 and control = 70). The data collection tool consisted of demographic data, menopause rating scale, international physical activities questionnaire, and TTM questionnaire (stages of change, processes of change, decision-making balance, and self-efficacy) that were completed before and two months after intervention. For intervention group, the educational program was implemented during 5 sessions of 50–55 min. Data were analyzed by SPSS 25 and by using Chi-square test, independent *t*-test, and paired *t*-test.

**Results:**

After intervention, the mean scores of physical symptoms are sleep problems, muscle problems and discomfort, psychological symptoms, depression, irritability, and anxiety of intervention group decreased significantly post intervention (*p* < 0.001). Analysis showed that psychological symptoms, mental fatigue, somatic, and urogenital symptoms, and vaginal dryness increased 2 months after the intervention. Also, the mean score of cons, pros, decision balance, self-efficacy, consciousness raising, dramatic relief, environmental reevaluation, and self-reevaluation have significant changed in intervention group (*p* < 0.05). In addition, the top percent of progress can be seen in contemplation (31. 5%).

**Conclusion:**

This study showed the effectiveness of educational intervention based on TTM to reduce menopausal symptoms and promote physical activity in postmenopausal women.

## 1. Introduction

Menopause is a biological stage and a natural and inevitable part of women's lives [1]. Due to the growing aging population, the number of postmenopausal women is steadily increasing [2]. Menopause causes short-term changes and long-term complications that can major impact women's general health, including physical, genitourinary, and psychological symptoms. It also affects different biological, psychological, and social aspects of women's lives [1–5]. Many studies showed that women experience at least one menopausal symptom [6–8]. Most women spend a third of their lives after menopause. So, quality of life over a long period will be a major concern [9, 10].

Physical activity as a corrective factor can be effective in improving the quality of life. Evidence shows that increasing the level of physical activity during menopause can help reduce psychological and social symptoms. [11–13]. Also, physical activity has a protective effect against many chronic diseases, including depression and anxiety [14]. In addition, the results of a study have shown that physical activity can be effective on symptoms related to menopause such as urogenital system, insomnia, and hot flashes [15]. Although menopausal symptoms have a significant impact on women's quality of life and physical activity has been able to reduce or eliminate these symptoms, little research has been done on ways to enhance physical activity in this population [12, 16]. Numerous studies have shown that physical activity without any side effects can decrease the symptoms of menopause [11, 17, 18]. The results of the national review stated an inadequate physical activity rate in the Iranian population of 39.1 percent. In addition, in the global report, Iran was also moderate to high among countries with insufficient physical activity [19, 20].

Knowledge about menopause, different symptoms of menopause, ways to reduce these symptoms is needed to adapt during this period, because it is a phase of life and an inevitable event. Various studies have shown that women have insufficient knowledge about menopause [21]. In our country, most women's health services are limited to prenatal care and family planning, and other women's needs, including menopause problems, have been neglected [22, 23]. In general, women's knowledge and practice about menopausal issues in Iran are low and this issue requires planned interventions [24, 25]. Because education is essential for community health, all women who experience menopause should be educated [5]. Implementing a structured training program can be an effective strategy to increase women's knowledge about menopausal symptoms and their management [3].

**Figure 1:**
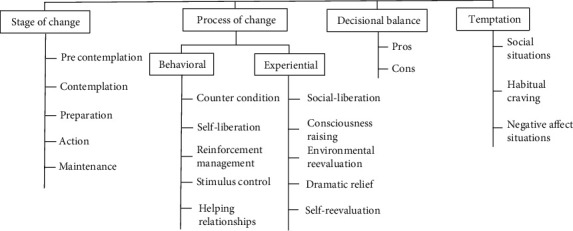
Schematic view of the transtheoretical model (TTM) constructs [56].

Since most postmenopausal women have good access to health centers, it seems that the most appropriate and accessible way to improve their health is to pay attention to menopause-related issues by health care providers and to hold training and counseling classes in the field of menopause with a health promotion approach [26]. Today, it is believed that behavior change is not an easy and fast process and with the useful use of behavior change theories, the effectiveness of education can be increased [27, 28]. One of the most widely used models for planning effective educational interventions is the transtheoretical model (TTM), which was first introduced by Prochaska and Diclemente [29, 30]. The steps of this model are shown in Figure 1.

As far as we know, according to the available evidence and the studies conducted in Iran and the world, there are few theory-based intervention programs to improve the symptoms of menopause and increasing the level of physical activity among postmenopausal women is felt. So, the aim of this study is to evaluate the application of the transtheoretical model in improving physical activity behavior to decrease menopausal symptoms in Pasargad City, Iran.

## 2. Materials and Methods

### 2.1. Study Design and Participants

This quasi-experimental study was performed on women 45 to 59 years old in 2020 in Pasargad City, Fars Province located in southwest Iran. The sample size was determined by using G^∗^Power software version 3 based on the previous study [31], with *B* = 0.95, *α* = 0.05, and considering the 30% drop in the participants, 70 people were considered for each group of intervention and control and totally 140.

**Figure 2:**
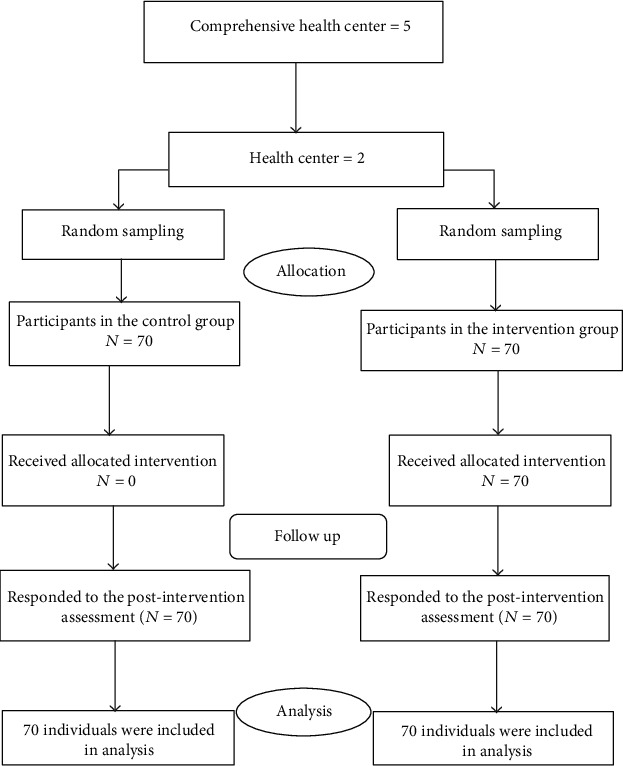
Consort flow chart of the participants in the study.

Participants were selected by selected multistage cluster sampling. Pasargad governmental comprehensive health center covers five health care centers. First, out of these five health centers, we choose two centers that had the most attended postmenopausal women. Then, one center was randomly assigned to the intervention group and one center was randomly assigned to the control group. Finally, based on the list of postmenopausal and eligible women, 70 women were randomly assigned to the intervention group and 70 women to the control group.  Figure 2 presents the study consort flow chart diagram.

The patients who were not willing to participate and have a medical prohibition for physical activities, were not on hormone drugs, did not take hormonal treatment, and who responded to the questionnaire incompletely were excluded from the study. The inclusion criteria were the women were 45-59 years old, living in the study area and not suffering from certain diseases, and disorders that cause changes in lifestyle or physical activity. The phone numbers of registered elders were extracted from their records in the health centers. Then, the trained staff contacted them by phone and introduced themselves. Besides, they explained the aims of the study and invited them to come to each health center which they selected if they were interested in participating in the study. Also, they were given the phone numbers of the executive team for any questions about this study. A face-to-face and personal interview was done with each of the participants. The time to fill out the questionnaire was between 20-30 minutes.

The data collection tools in this study were five self-reported questionnaires and the interview was completed if necessary, including the following:

Demographic variables questionnaire: this questionnaire included information such as age, marital status, level of education, employment status, smoking (hookah, cigarettes), suffering from disease, taking medication, and housing situation.

Menopause Rating Scale (MRS): Menopause Rating Scale was designed by Heinemann et al. [32]. The Persian version of this questionnaire has been confirmed by Jahangiry et al. [33] was used to the severity of menopausal complaints, and to determine the pattern of menopausal symptoms and their effects on health-related quality of life. It comprises 11 items consisting of three dimensions: somatic symptoms (4 items), psychological symptoms (4 items), and urogenital symptoms (3 items) with responses on a five-point Likert scale with 0 = *none*, 1 = mild, 2 = moderate, 3 = severe, and 4 = very severe. The total MRS score was determined by the sum of the scores of each subscale. The values upper 8 (somatic), 6 (psychological), 3 (urogenital), and 16 (total MRS) were defined as severe scores [34]. Cronbach's alpha was .931 [33].

International Physical Activity Questionnaire (IPAQ): physical activity intensity levels were measured using the 7-question International Physical Activity Questionnaire [35]. According to the designed questionnaire, the amount of intense, moderate and walking physical activity in the last 7 days is determined. Physical activity was classified into three levels (low physical activity, sufficient physical activity, and high physical activity). Physical activity level (week/min-METs) less than 600 was classified as low physical activity, between 600-3000 as moderate physical activity and more than 3000 in high physical activity group [36].The Persian version of IPAQ short form and its validity and reliability had been evaluated and confirmed by Moghaddam et al. [37].

TTM questionnaire: it was a researcher-made questionnaire that of 41 items. The first item was a 5-choice question to determine the stages of change in terms of physical activity behaviors that the definition of these steps in terms of physical activity behavior was developed by Marcus et al. [38]. In this questionnaire, participants were asked if they do at least 30 minutes of moderate physical activity (such as brisk walking) for 3-5 more days a week. According to the participants' answers, they were placed in one of the stages of precontemplation, contemplation, preparation, action, and maintenance. In this study, the Cronbach *α* was 0.92. The item 2 to item 18 to determine processes of change included cognitive and behavioral items which were rated on a 5-point scale from 0 (completely disagree) to 4 (completely agree). Its content validity was confirmed by a group of health promotion specialists (*n* = 10). Also, CVR = 0.73 and CVI = 0.86 confirm the validity of this tool. Cronbach's alpha was 0.87. The items 19-28 to determine the self-efficacy which was rated on a 5-point scale of zero (completely disagree) to (completely agree) to 4 scores given to each item, respectively. Also, Cronbach's alpha coefficient of the self-efficacy construct questionnaire was calculated to be *α* = 0.72. Rest of item was assessing decision-making balance includes the benefits and hindrances of physical activity and is rated from 0 (completely agree) to 4 (completely disagree). We calculated the reliability of the decision-making balance *α* = 0.90.

Before the intervention, the questionnaire was completed and the participants were placed in one of the stages (precontemplation, contemplation, preparation, action, and maintenance). Then, the intervention program was implemented, with the following activities.

In this study, the intervention was educational in the field of physical activity training for postmenopausal women during 5 sessions (theoretical and practical) and each session lasted an average of 60 minutes. The practical sessions included doing physical activities that they did aerobic, stretching, normal walking with full inhalation and exhalation, and walking by counting steps per minute which three days a week. Also, specific educational content was set for each session and different educational methods were used to increase learning and participation of the participants, such interactive lectures, group discussions, educational clips, posters, photos, and PowerPoint. The first session was dedicated to the introduction of physical activities (along with educational slides) and also the dangers of a sedentary life, and the benefits of physical activity. (increasing alertness). In the second session (dramatic relief) was about menopausal symptoms and the benefits of physical activity in reducing menopausal symptoms were expressed for 20 minutes, and aerobic, balance, and stretching exercises were performed for 40 minutes in the park. In the third and fourth sessions (environmental reevaluation, self-reevaluation, and bolstering self-efficacy), low-cost or free options for physical activity were discussed. Also, places where it is possible to do physical activity at a low cost were introduced. In addition, new ways to do physical activity were suggested. Verbal encouragement was also used to persuade participants for 20 minutes then the correct way of walking and respiration were educated in a park for 40 minutes at last the participants' questions on the taught topics were answered. In the last session, 25 minutes of walking was done at a speed of 80 steps per minute under monitoring a trainer and the cooperation of the participants, and the way of cooling down was instructed for 30 minutes, finally, the exercises at home were given to the participants (self-liberation).

It is noteworthy that before the educational intervention, the necessary explanations about the research were given to the participants and written consent was obtained from them. Participants were also assured that their information would remain confidential. Also, in order to maintain ethical considerations, after the posttest, a training package containing pamphlets and booklets that was consist of key and main points was provided to the control group.

### 2.2. Statistical Analysis

Data was entered into the IBM SPSS statistics software version 25 [39] and the accuracy of data entry was checked by randomly selecting the data from the software and matching them with the related questionnaires. Demographic variables were compared between two groups with the Chi-square test. The independent *t*-test was used to compare the changes between the two groups of intervention and control. To evaluate the effect of the intervention paired *t*-test was utilized. To compare the TTM construct, in each group, Paired *t*-test was used. *P* value lower than 0.05 was considered statistically significant.

## 3. Results

There were no statistically significant differences between the two groups before the intervention in demographic variables. Based on the results, the mean age of women who participated in the study was 52.95 ± 4.77 years for the intervention group and 52.55 ± 4.52 years for the control group (*P* = 0.612). The highest frequency of level of educated postmenopausal women in both control and intervention groups is lower than twelfth-grade at 61 (87.10) and 64 (91.40), respectively, (*P* = 0.586). In terms of employment status, 62 (88.60%) of participants in the intervention group and 60 (85.70%) in the control were unemployed (*P* = 0.801). Comparing demographic variables and medical backgrounds of participants in intervention and control groups are shown in Table 1.

Table 2 shows that there were significantly different between pre and postintervention physical symptoms, sleep problems, muscle problems, discomfort, psychological symptoms, depression, irritability, and anxiety in the intervention group and it showed these problems decreased after the intervention. The most important and biggest reduction in the intervention group can be seen in physical symptoms which are reduced by 0.73. However, there were significant differences between pre and postintervention of psychological symptoms, depression, and irritability of the control group and it showed these complications increased in this group during the time of intervention. Moreover, an independent *t*-test showed that psychological symptoms, mental fatigue, somatic, and urogenital symptoms, and vaginal dryness had significant differences in both groups (intervention and control) before and after the intervention.

**Figure 3:**
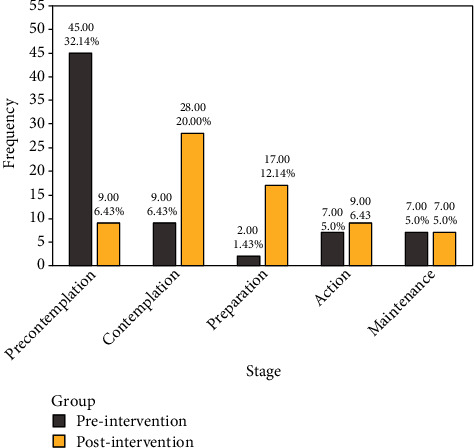
Frequency of each stage of change in both pre and postintervention.

Figure 3 illustrates the frequency of five stages in both pre and postintervention. Besides, the most stage change was shown in precontemplation and contemplation which received 45 to 9, 9 to 28 in pre and postintervention, respectively. Further, it depicted the participants who in maintenance did not change their stage.

Table 3 shows the mean of constructs cons, pros, decision balance, self-efficacy, consciousness raising, dramatic relief, environmental reevaluation, self-reevaluation, counterconditioning, helping relationships, stimulus control, social liberation, and self-liberation during 2 periods of preintervention, and 2 months after the intervention. Paired *t*-test has shown that cons, pros, decision balance, self-efficacy, consciousness raising, dramatic relief, environmental reevaluation, and self-reevaluation have significant changes (*P* < 0.05). The most important and biggest increase in the intervention group can be seen in decision balance which is improved by 2.77.

## 4. Discussion

The present study is aimed at determining the effect of educational intervention based on the transtheoretical model (TTM) on physical activity and menopausal symptoms of female clients of the health center in Pasargad City. After two months of intervention, the study revealed that physical symptoms, sleep problems, muscle problems and discomfort, psychological symptoms, depression, irritability, and anxiety had a significant decrease in the intervention group. This is consistent with the results of other studies [9, 40, 41]. Besides, Paired *t*-test has shown that cons, pros, decision balance, self-efficacy, consciousness raising, dramatic relief, environmental reevaluation, and self-reevaluation have significant changes in the intervention group during this period. The changes in stages in the intervention group had shown progress of 15.57% and 10.71% in the contemplation and preparation stages, respectively. Consistent with our study a study reported that cognitive-behavioral training based on self-efficacy can decrease stress [42]. Further, Malekshahi et al. resulted that the construct of self-efficacy had the highest predictive power of preventive behavior. The results showed that self-efficacy among the constructs of the TTM was the only predictive construct for osteoporosis prevention behavior [43]. A study done by Koyuncu et al. [44] has also shown that there was a decrease in the somatic and psychological subdimensions of the menopausal symptom evaluation scale and in the total score which is in line with our results and positive changes were observed in the levels of knowledge about menopause. Yazdkhasti et al. [45] noticed empowerment of menopausal women will guarantee their health during the last third of their life. It will also help them benefit from their final years of reproductive life. Moon et al. [46] demonstrated clinical implications in terms of targeting women who are more at risk and offering nonhormonal treatment options, to help women to develop self-reevaluation strategies for coping with menopausal symptoms in line with our findings. Rindner et al. found the intervention group experienced a slight reduction in symptoms while the control group mostly experienced the opposite [47]. This study indicated a reduction in anxiety and depression scores over time age which is similar to other findings [48, 49]. Results also demonstrated reduced vasomotor symptoms and sexual dysfunction.

Results also indicated reduced vasomotor symptoms and sexual dysfunction. They showed that lifestyle intervention embedded within a wellness framework has the potential to reduce menopausal symptoms and improve quality of life in midlife women thus potentially enhancing health and well-being in women. These findings were consistent with the studies by Wong et al. [50], and Esposito et al. [51]. Our findings showed that education intervention reduces psychological symptoms of depression and anxiety but does not reduce other somatic, urogenital, and vasomotor symptoms. This lack of change in the scores of physical, urogenital, and vasomotor symptoms can be due to hormonal reasons and metabolic syndrome [52, 53]. As result of Kaya et al. shown in the study, testosterone and progesterone levels have an effect on psychological symptoms, urogenital symptoms and the severity of menopause symptoms [53]. However, Daley et al. [40] have recommended that women be advised to consider aerobic exercise as a treatment for vasomotor menopausal symptoms. Kim et al. [54] notified a moderate level of physical activity was associated with reduced psychosocial and physical menopause symptoms in premenopausal Korean women which is similar to our findings in our study. Mirzaiinjmabadi et al. showed that exercise was effective in relieving somatic and psychological symptoms, including depression and anxiety [55]. In addition, McAndrew et al. suggested that physical activity participation is associated with lower general symptom reporting as opposed to specifically impacting menopause symptoms. Moreover, exercise self-efficacy mediates the relationship between physical activity and general menopause symptoms in line with our findings [49].

## 5. Limitation and Strength

The present study is a theory-based study and a randomized controlled trial. Another strong point of this study is the use of MRS valid tool to check the symptoms of menopause. In addition, the results of this study and intervention based on this theory can be generalized for all women. Although the current research has several strengths but the recall bias in relation to recalling the questions related to the MRS questionnaire is one of the limitations of this study, because the study subjects expressed their menopausal symptoms during the past month.

## 6. Conclusion

The findings of this study can be served by authorities as a base for educational interventions in behavioral changes by increasing physical activity to increase menopausal acceptance in postmenopausal women and can be effective to decrease menopausal symptoms in improving the quality of life and reducing the treatment and medical care costs. Furthermore, life satisfaction may be enhanced through the improvement of mental and physical parameters.

## Figures and Tables

**Table 1:** Comparing demographic variables and medical backgrounds of participants in intervention and control groups.

Variable	Group		*P* value
	Intervention(*n* = 70)	Control(*n* = 70)	
	*N* (%)/*mean* ± *SD*	*N* (%)/*mean* ± *SD*	
Age	52.95 ± 4.77	52.55 ± 4.52	*P* = 0.612
*Married status*			
Single	8(11.4)	14(20.0)	*P* = 0.245
Married	62(88.60)	56(80.0)	
*Education*			
< twelfth grade	64(91.40)	61(87.10)	*P* = 0.586
> twelfth grade	6(8.60)	9(12.90)	
*Suffer from disease*			
Yes	42(60.0)	36(51.40)	*P* = 0.395
No	28(40.0)	34(48.60)	
*Taking medication*			
Yes	42(60.0)	35(50.0)	*P* = 0.308
No	28(40.0)	35(50.0)	
*Smoking(cigarettes, hookahs)*			
Yes	4(5.70)	4(5.70)	*P* = 1.000
No	66(94.30)	66(94 30)	
*Employment status*			
Un employed	62(88.60)	60(85.70)	*P* = 0.801
Employed	8(11.40)	10(14.30)	
*Housing situation*			
Personal	63(90.0)	65(92.90)	*P* = 0.397
Leased	7(10.0)	4(5.70)	

**Table 2:** Comparison of three categories of menopausal symptoms and their components at preintervention and 2 months after intervention in intervention and Control groups.

Variable	Group	Preintervention	After intervention	Paired *t*-test
		*M* ± *SD*	*M* ± *SD*	
*Physical symptoms*	Intervention	7.07 ± 2.66	6.34 ± 2.55	*P* < 0.001
	Control	6.07 ± 3.11	6.32 ± 3.15	*P* = 0.126
Independent sample-test		*P* = 0.518	*P* = 0.043	
*Flushing*	Intervention	2.12 ± 1.43	2.08 ± 2.42	*P* = 0.182
	Control	1.87 ± 1.31	1.95 ± 1.30	*P* = 0.182
Independent sample-test		*P* = 0.578	*P* = 0.271	
*Heart disease*	Intervention	1.10 ± 1.05	1.02 ± 1.00	*P* = 0.058
	Control	0.87 ± 1.02	0.94 ± 1.01	*P* = 0.058
Independent sample-test		*P* = 0.618	*P* = 0.194	
*Sleep problems*	Intervention	1.55 ± 1.30	1.27 ± 1.20	*P* = 0.002
	Control	1.22 ± 1.14	1.31 ± 1.16	*P* = 0.135
Independent sample-test		*P* = 0.831	*P* = 0.115	
*Muscle problems and discomfort*	Intervention	2.28 ± 1.09	1.95 ± 1.05	*P* = 0.001
	Control	2.10 ± 1.27	2.11 ± 1.29	*P* = 0.765
Independent sample-test		*P* = 0.432	*P* = 0.365	
*Psychological symptoms*	Intervention	6.62 ± 3.79	5.74 ± 0.32	*P* = 0.001
	Control	4.95 ± 3.52	5.21 ± 3.63	*P* = 0.028
Independent sample-test		0.005	*P* = 0.008	
*Depression*	Intervention	1.34 ± 1.30	1.14 ± 1.15	*P* = 0.012
	Control	1.00 ± 1.16	1.08 ± 0.20	*P* = 0.013
Independent sample-test		*P* = 0.775	*P* = 0.104	
*Irritability*	Intervention	1.32 ± 1.17	1.17 ± 1.06	*P* = 0.011
	Control	1.15 ± 1.01	1.21 ± 1.06	*P* = 0.045
Independent sample-test		*P* = 0.812	*P* = 0.358	
*Anxiety*	Intervention	1.87 ± 1.12	1.47 ± 0.92	*P* < 0.001
	Control	1.42 ± 1.09	1.51 ± 1.08	*P* = 0.083
Independent sample-test		*P* = 0.802	*P* = 0.020	
*Mental fatigue*	Intervention	2.08 ± 1.28	1.97 ± 1.27	*P* = 0.060
	Control	1.37 ± 1.26	2.04 ± 2.25	*P* = 0.151
Independent sample-test		*P* = 0.010	*P* = 0.001	
*Somatic, and urogenital symptoms*	Intervention	2.64 ± 2.60	2.49 ± 2.42	*P* = 0.180
	Control	2.01 ± 2.28	2.04 ± 2.28	*P* = 0.321
Independent sample-test		0.015	*P* = 0.080	
*Sexual problems*	Intervention	0.84 ± 1.25	0.80 ± 1.25	*P* = 0.083
	Control	0.58 ± 1.09	0.60 ± 1.10	*P* = 0.321
Independent sample-test		*P* = 0.312	*P* = 0.200	
*Vaginal dryness*	Intervention	1.14 ± 1.31	1.10 ± 1.32	*P* = 0.320
	Control	0.72 ± 0.99	0.74 ± 1.00	*P* = 0.321
Independent sample-test		*P* = 0.045	*P* = 0.037	
*Bladder problems*	Intervention	0.75 ± 0.95	0.70 ± 0.84	*P* = 0.321
	Control	0.70 ± 1.01	0.74 ± 1.05	*P* = 0.321
Independent sample-test		*P* = 1.000	*P* = 0.732	

**Table 3:** Comparison of mean scores of TTM constructs before and 2 months after educational intervention in intervention and control groups.

Trans theoretical construct	Before intervention	Two months after intervention	Paired *t*-test
*Cons*			
Intervention group	14.41 ± 5.07	14.02 ± 4.78	0.035
Control group	16.01 ± 4.78	16.22 ± 5.19	0.808
Independent sample-test	0.69	0.013	
*Pros*			
Intervention group	21.25 ± 5.24	23.62 ± 4.77	0.008
Control group	21.21 ± 4.79	21.24 ± 4.59	0.971
Independent sample-test	0.960	0.082	
*Decision balance*			
Intervention group	6.83 ± 4.22	9.60 ± 4.04	0.022
Control group	5.20 ± 4.14	5.01 ± 4.26	0.794
Independent sample-test	0.022	0.026	
*Self-efficacy*			
Intervention group	25.00 ± 6.63	27.64 ± 6.25	0.017
Control group	24.42 ± 6.36	24.25 ± 6.25	0.873
Independent sample-test	0.064	0.002	
*Consciousness raising*			
Intervention group	5.30 ± 2.24	7.08 ± 1.70	*p* < 001
Control group	5.97 ± 1.96	6.15 ± 1.79	0.560
Independent sample-test	0.062	0.002	
*Dramatic relief*			
Intervention group	5.85 ± 1.82	6.92 ± 1.40	*p* < 001
Control group	6.12 ± 1.99	6.20 ± 1.88	0.828
Independent sample-test	0.410	0.011	
*Environmental-reevaluation*			
Intervention group	3.00 ± 1.14	3.72 ± 0.866	*p* < 001
Control group	3.22 ± 1.10	3.24 ± 1.08	0.939
Independent sample-test	0.231	0.004	
*Self-reevaluation*			
Intervention group	11.94 ± 3.46	13.97 ± 2.45	*p* < 001
Control group	11.80 ± 3.49	12.18 ± 3.26	0.501
Independent sample-test	0.809	*p* < 001	
*Self-liberation*			
Intervention group	5.92 ± 1.85	6.52 ± 1.93	0.063
Control group	5.78 ± 1.86	5.81 ± 1.86	0.928
Independent sample-test	0.650	0.028	

## Data Availability

Data used in the analysis as well as all programs used for the analysis may be obtained by contacting the corresponding author on reasonable request.

## Abbreviations

TTM: Trans theoretical Model
